# The Feline Immunodeficiency Virus Envelope Signal Peptide Is a Tetherin Antagonizing Protein

**DOI:** 10.1128/mbio.00161-23

**Published:** 2023-03-16

**Authors:** James H. Morrison, Eric M. Poeschla

**Affiliations:** a Division of Infectious Diseases, University of Colorado Anschutz Medical Campus, Aurora, Colorado, USA; The University of Texas at Austin; Rutgers-Robert Wood Johnson Medical School

**Keywords:** tetherin, HIV-1, FIV, restriction factor, accessory protein, innate immunity, signal peptide, signal sequence, leader sequence, BST2

## Abstract

Signal peptides are N-terminal peptides, generally less than 30 amino acids in length, that direct translocation of proteins into the endoplasmic reticulum and secretory pathway. The envelope glycoprotein (Env) of the nonprimate lentivirus feline immunodeficiency virus (FIV) contains the longest signal peptide of all eukaryotic, prokaryotic, and viral proteins (175 amino acids), yet the reason is unknown. Tetherin is a dual membrane-anchored host protein that inhibits the release of enveloped viruses from cells. Primate lentiviruses have evolved three antagonists: the small accessory proteins Vpu and Nef, and in the case of HIV-2, Env. Here, we identify the FIV Env signal peptide (Fsp) as the FIV tetherin antagonist. A short deletion in the central portion of Fsp had no effect on viral replication in the absence of tetherin, but severely impaired virion budding in its presence. Fsp is necessary and sufficient, acting as an autonomous accessory protein with the rest of Env dispensable. In contrast to primate lentivirus tetherin antagonists, its mechanism is to stringently block the incorporation of this restriction factor into viral particles rather than by degrading it or downregulating it from the plasma membrane.

## INTRODUCTION

The evolution of host antiviral factors has selected for reciprocal evolution of viral countermeasures, which can act through passive avoidance or direct antagonism. Tetherin (BST-2) is a type I interferon (IFN) inducible protein that forms homodimers, and directly links newly formed HIV-1 particles and the plasma membrane through its transmembrane domain and a C-terminal GPI-anchor (1, 2). This attachment function prevents viral particle release from infected cells and can lead to virus internalization, degradation via endosomal/lysosomal pathways, and induction of NF-κB-dependent proinflammatory responses in the infected cell (3–5).

The importance of tetherin evasion for primate lentiviruses is indicated by their nearly ubiquitous encoding of antagonists. Three different such proteins have been described. SIVs of *Cercopithecus* genus primates (SIVgsn, SIVmus, and SIVmon) and HIV-1 counteract tetherin with the accessory protein Vpu (6). SIVcpz, the proximate precursor to HIV-1, shares common ancestry with cercopithecine SIVs, yet utilizes Nef to counteract tetherin (6). Other SIVs also utilize Nef to antagonize tetherin, including SIVsmm, the virus proximately ancestral to HIV-2 (7–9). A small deletion in the cytoplasmic tail of human tetherin prevents Nef binding (6). As SIVsmm transitioned to Homo sapiens to become HIV-2, it gained tetherin antagonizing function in Env (10), whereas the HIV-1 subgroups, which arose from independent cross-species transmission events, vary in this regard. Nonpandemic HIV-1 group O strains lack an efficient anti-tetherin mechanism, but pandemic HIV-1 group M strains evolved a Vpu capable of counteracting tetherin (6). These primate lentiviral proteins all act by functionally depleting tetherin from the plasma membrane via intracellular sequestration or endocytosis and lysosomal degradation of the protein (8–10).

For nonprimate lentiviruses, much less is known about viral interaction with and evasion of tetherin. Their accessory gene repertoires are apparently more limited and Vpu and Nef are found only in primate lentiviruses. For that matter, no new lentiviral accessory genes have been identified for decades. However, cat and dog tetherin proteins both restrict HIV-1 and feline immunodeficiency virus (FIV) particle budding, and both carnivore proteins are antagonized by FIV Env (11). While this situation superficially resembles the antagonism of human tetherin by HIV-2 Env, major differences were observed that suggest different mechanisms. Unlike primate lentiviral antagonists, we found that the FIV Env mechanism does not require processing of Env into its surface unit (SU) and transmembrane (TM) domains (11). It also shields the budding particle without downregulating plasma membrane tetherin, and does not rescue noncognate (e.g., HIV-1) virus budding (11). Here, we explored the mechanism of FIV tetherin antagonism further and determined that it derives specifically from the signal peptide, which functions autonomously from Env, and acts to prevent particle incorporation.

## RESULTS

### Mapping of determinants of tetherin antagonism.

We previously reported that the envelope glycoprotein (Env) of FIV counteracts restriction of this virus by both domestic cat and dog tetherin proteins when these were either transiently or stably expressed in cell lines (11). Here, we also examined primary domestic cat tissues for tetherin protein expression. Levels were undetectable by immunoblotting in normal domestic cat peripheral blood mononuclear cells (PBMCs), but the protein was induced by IFN-α (Fig. S1A). Some but not all immortalized feline cell lines were similarly inducible, e.g., fibroblastic K-ER cells, while CrFK cells were not (Fig. S1A). A variety of primary domestic cat tissues had variable but generally detectable levels of tetherin gene transcripts (Fig. S1B). Immunoblotting revealed comparatively high levels in spleen, lymph node, heart, and lung, with lesser protein levels observed in tonsil, brain, kidney, and gut tissues (Fig. S1B and C). These results suggest that FIV may encounter tetherin in cell type or induction-specific contexts *in vivo*.

10.1128/mbio.00161-23.1 FIG S1 Tetherin expression in domestic cat cells and tissues. (A) Domestic cat cell lines, primary cat PBMCs and human 293T cells that stably express domestic cat tetherin were treated with 2 ng/mL human IFN-α for 24 h. Cell lysates were immunoblotted with mouse anti-cat tetherin and goat anti-mouse IgG-HRP. (B) Absolute copies of *Tetherin* and *GAPDH* transcripts were quantified by qPCR (*n* = 1 to 3 per tissue, as available). (C) Lysates of various tissues from a healthy adult cat were assessed for tetherin expression by immunoblotting with mouse anti-cat tetherin and goat anti-mouse IgG-HRP. Download FIG S1, TIF file, 2.5 MB. Copyright © 2023 Morrison and Poeschla. 2023 Morrison and Poeschla. https://creativecommons.org/licenses/by/4.0/ This content is distributed under the terms of the Creative Commons Attribution 4.0 International license.

**FIG 1:**
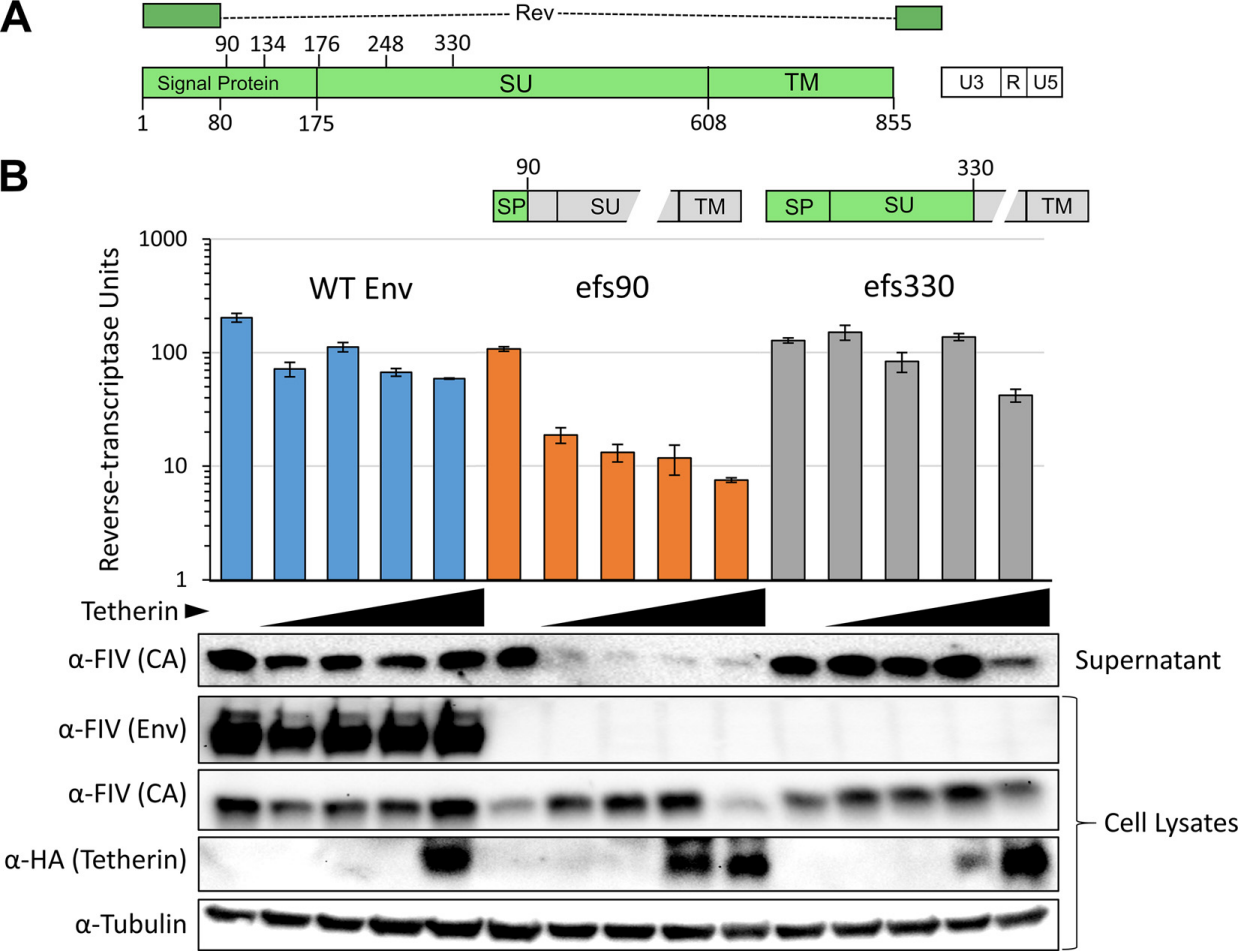
SU and TM are dispensable for tetherin antagonism. (A) Diagram of FIV *Env* gene. Numbering indicates amino acids from the Env initiator methionine, which is shared with Rev. (B) 293T cells were co-transfected with increasing amounts of Tsin HA-Tetherin (0, 62.5, 125, 250, 500 ng) and 1,000 ng pFIVC36 proviral constructs (pCT-C36^A+^ based) encoding an intact Env (blue bars), or an Env frameshift (efs) mutation at amino acid 90 (orange bars) or at amino acid 330 (gray bars). An empty vector (Tsin IRES-puro) was used to bring all transfections to 1,500 ng total. Diagrams indicate intact (green) or disrupted (gray) Env subunits. Cell lysates and 0.45 μm filtered supernatants were harvested 48 h after transfection and immunoblotted with the indicated primary antibody and a corresponding HRP-conjugated secondary antibody. Supernatants were analyzed for reverse-transcription activity and shown as average +/− standard deviation of three technical replicates. Experiment was repeated four times and a representative example is shown.

FIV Env protein’s anti-tetherin activity is independent of proteolytic processing of Env into SU and TM domains (11). FIV Rev and Env have the same initiator methionine codon but are differentiated by alternative splicing; therefore, Rev and Env share the first 80 amino acids (Fig. 1A). To identify the minimal components of Env necessary to enable nascent FIV virion escape from tetherin-expressing cells, we constructed a series of Env frameshift (efs) mutants that progressively truncate the protein while leaving Rev intact. These were constructed in FIVC36, an infectious molecular clone that replicates to high levels *in vivo* and causes AIDS (12). Antagonism of tetherin was determined by quantifying FIV particles in cell supernatants following co-transfection of the FIV proviral construct and tetherin plasmids (Fig. 1B). Co-expression of an Env-intact FIV with increasing amounts of feline tetherin resulted in a modest reduction in reverse transcriptase (RT) activity and capsid (CA) in supernatants (Fig. 1B, blue bars and supernatant immunoblot). Introduction of a frameshift in the signal peptide of Env (amino acid 90) resulted in a virus that was significantly more sensitive to the presence of tetherin, with viral budding decreased in proportion to feline tetherin input (Fig. 1B, orange bars and immunoblot). In contrast, termination of Env in mid-SU, at residue 330, reverted the phenotype, whereby again FIV was only modestly affected by tetherin co-expression (Fig. 1B, gray bars).

**FIG 2:**
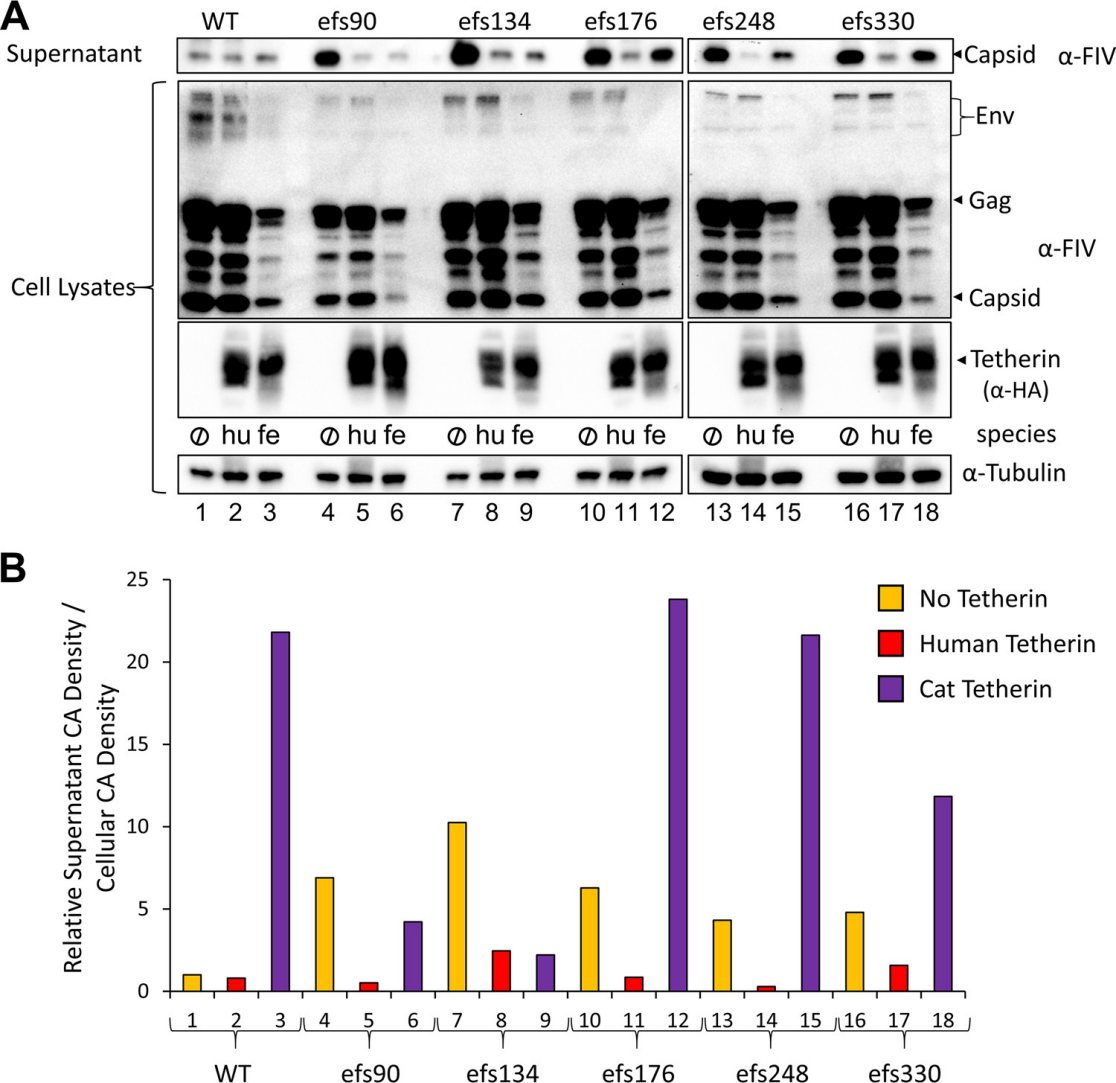
Effects of Env frameshift mutations on feline tetherin restriction. (A) 293T cells having the indicated tetherin stably expressed under puromycin selection were transfected with 1.5 μg of the indicated pC36 proviral construct. Numbering indicates the last intact Env amino acid before early protein termination due to a frameshifting mutation. Cell lysates and supernatants were harvested and analyzed 48 h after transfection as in Fig. 1. (B) Bands corresponding to supernatant and cell lysate FIV capsid from panel A were density quantified using ImageJ. The ratio of the supernatant to intracellular capsid bands were calculated and normalized to WT FIVC36 from cells without tetherin (lane 1, assigned a value of 1). Lane numbers are the same as panel A. Experiment was repeated four times and a representative example is shown.

To further map the virus-rescuing activity, a set of three additional truncations was made by introducing frameshifts within the N-terminal portion of Env (efs134, efs176, and efs248). These, plus the initial efs90 and efs330 viruses, were tested for tetherin susceptibility, this time using cells that stably express either human or feline tetherin (Fig. 2A). As expected, each viral variant expressed equivalent intracellular levels of FIV core proteins, budded in the absence of tetherin, and was blocked from budding by human tetherin (Fig. 2A). Absence of the Env SU or TM domains did not significantly affect the ratio of intracellular Gag/CA to budded particles (efs176, efs248, and efs330). It was only when the signal peptide of Env was truncated (efs134 and efs90) that a loss of FIV budding was observed in cells that express domestic cat tetherin; again, human tetherin restriction was not abrogated (Fig. 2A). Quantification of immunoblot densities confirmed that when normalized for intracellular capsid expression levels, feline tetherin was effective at blocking budding of the efs90 and efs134 mutants, whereas wild-type FIVC36 and the efs mutants retaining at least the first 176 amino acids of Env were resistant to feline tetherin and even had higher ratios of released to intracellular capsid compared to control cells lacking tetherin (Fig. 2B).

Considering these data, we hypothesized that the FIV Env signal peptide (Fsp) is the necessary factor that mediates feline tetherin antagonism. We further hypothesized that it is sufficient (autonomously acting). Signal peptides, also known as leader sequences or signal peptides, are N-terminal peptides that were discovered by Blöbel and colleagues (13, 14) to act as cellular “zip codes” that direct targeted translocation of newly synthesized proteins into the endoplasmic reticulum (ER) lumen in a signal recognition particle-dependent manner. Analogous subcellular targeting motifs, e.g., for mitochondria have also been described (15). For enveloped viruses, signal peptides are the predominant mechanism targeting viral surface proteins to the correct cellular compartment to enable proper particle incorporation. In both eukaryotes and prokaryotes, signal peptide lengths are generally very short, with a mean length of 23 ± 6 amino acids (16). Primate lentiviruses encode somewhat longer Env signal peptides, ranging between 19 and 45 amino acids in total length (Fig. S2). Remarkably, our database searches and literature reviews indicate that FIV encodes the longest known signal peptide in any eukaryotic or prokaryotic species, or in any virus (175 amino acids). Despite the reporting of this elongated signal peptide over 25 years ago (17, 18), the functional implications are unknown.

10.1128/mbio.00161-23.2 FIG S2 Signal peptides comparison. Signal peptide lengths reported are from citations below, manual assertion according to sequence analysis in the Uniprot database (54) or were previously analyzed using the PCgene program (35). (A) Lentiviral signal peptides were compared with other retroviruses with known additional function, including MMTV Rem (Rev-like vRNA nuclear export) (55), JSRV (posttranscriptional gene expression regulation) (34). Viruses are color-coded: nonprimate lentivirus (light blue), primate lentivirus (dark-blue), and betaretrovirus (green). (B) Human albumin, SARS-CoV-2 Spike, Ebola GP, HIV-2 Env, HIV-1 Env, and FIV Env signal peptides. Hydrophobic regions are shown in red font. Download FIG S2, TIF file, 2.0 MB. Copyright © 2023 Morrison and Poeschla. 2023 Morrison and Poeschla. https://creativecommons.org/licenses/by/4.0/ This content is distributed under the terms of the Creative Commons Attribution 4.0 International license.

### Fsp directs endoplasmic reticulum translocation.

**FIG 3:**
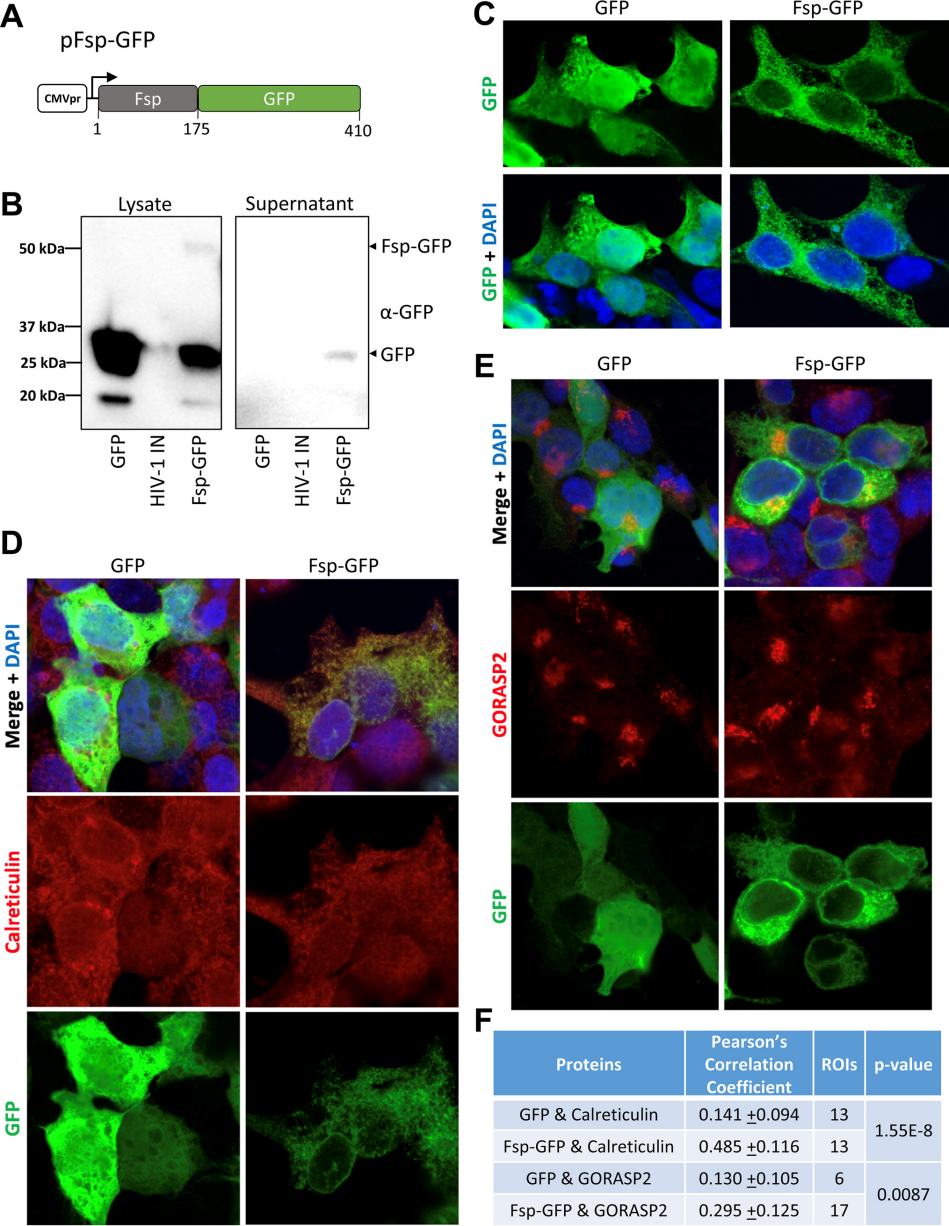
Fsp can direct protein translocation into the secretory pathway. (A) Diagram of the Fsp-GFP construct. (B) 293T cells were transfected with 1.5 μg of plasmids that express eGFP, myc-tagged HIV-1 integrase (negative control for blot), or Fsp-GFP (pEGFP-N1, HIV-1-IN-myc, pFsp-GFP) and cell lysates and 0.22 μm filtered supernatants were collected 48 h posttransfection and immunoblotted with mouse anti-GFP and goat anti-mouse HRP. (C to F) Confocal microscopy of 293T cells transfected with pEGFP-N1 or pFsp-GFP fixed 16 h posttransfection. Cells were mounted with ProLong Gold containing DAPI without additional staining (C) or were stained with rabbit anti-Calreticulin (D) or rabbit anti-GORASP2 (E) and Alexafluor 594 goat-anti-rabbit. (F) The ImageJ plugin Coloc2 was used to calculate Pearson’s Correlation Coefficient (+/− standard deviation) for GFP (GFP or Fsp-GFP) and Calreticulin or GORASP2. Individual regions of interest (ROIs) were drawn over individual transfected cells or small groups of cells prior to each analysis. A two-tailed *T*-test was performed to compare GFP and Fsp-GFP colocalization with the indicated cellular marker (*P*-value column).

To characterize the Fsp protein for localization properties and to test if it can fulfill traditional signal peptide functions in addition to acting as a tetherin antagonist, we expressed the signal peptide *in trans*, as a fusion to a virologically irrelevant, trackable protein (GFP; Fig. 3A). In immunoblots for GFP, although Fsp-GFP was detectable, we observed intracellular accumulation of predominantly free GFP (Fig. 3B, left), indicating that Fsp is efficiently cleaved from GFP at the signal peptidase cleavage site. Free GFP was also detectable in the filtered supernatant of Fsp-GFP-transfected but not GFP-transfected cells, indicating Fsp directed GFP translocation into the ER lumen and export via the secretory pathway (Fig. 3B, right). Furthermore, immunofluorescence experiments were strongly suggestive of localization in the ER for the cleaved GFP signal in Fsp-GFP expressing cells (Fig. 3C). Confirming this, GFP signal from Fsp-GFP transfected cells co-localized with the ER-resident protein calreticulin and, to a lesser degree, the Golgi apparatus marker GORASP2 compared with statistically weaker co-localization observed in GFP transfected cells (Fig. 3D to F). Cumulatively, the data confirm that the FIV Env N-terminal 175 amino acids act as a bona fide signal peptide to direct protein translocation.

### Deletion of a central Fess motif has no effect on viral replication in the absence of tetherin but severely impairs virion release in its presence.

**FIG 4:**
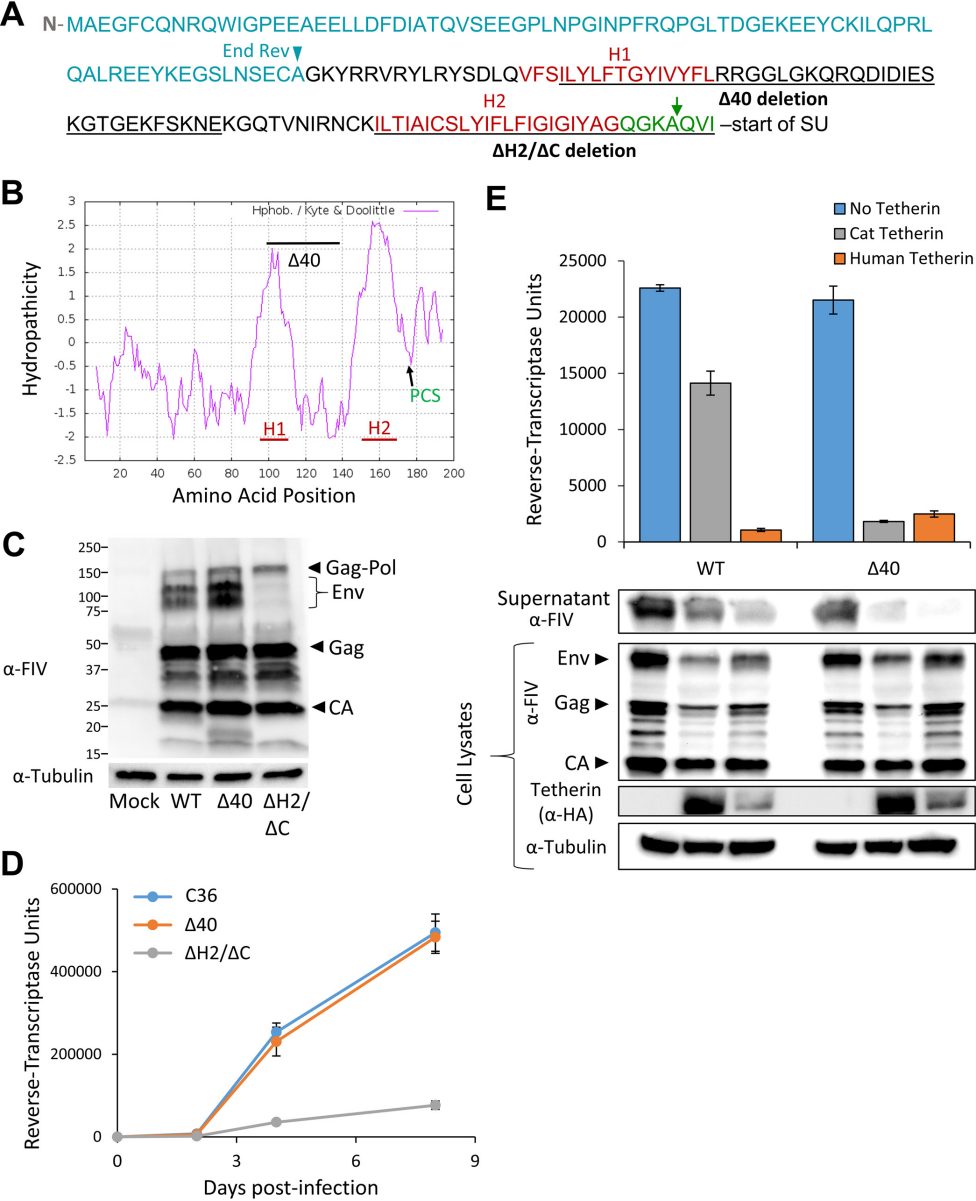
Fsp has separable roles in Env expression and tetherin antagonism. (A) Fsp amino acid sequence (FIV C36). Approximate hydrophobic regions H1 and H2 are indicated in red font, the C-region in green font, with a green arrow denoting the predicted signal peptidase cleavage site (18). The regions deleted in Δ40 and ΔH2/ΔC mutants of FIV C36 are underlined. Amino acids that are shared with Rev are in teal font. (B) FIVC36 Envelope amino acids 1 to 200 were plotted for hydropathicity using the Kyte and Doolittle model. H1: hydrophobic peak 1. H2, hydrophobic peak 2; PCS, predicted signal peptidase cleavage site. (C) 293T cells lacking tetherin were transfected with the indicated FIVC36 proviral constructs, and 48 h posttransfection cell lysates were harvested and immunoblotted with cat sera reactive to FIV. (D) CrFK cells stably expressing the FIV receptor CD134 were infected with the indicated viruses which were input normalized to reverse transcriptase content. Cells were washed twice 16 h postinfection and every other day 50 μL supernatant was collected for reverse transcriptase quantification. Spreading replication was performed twice and one experiment is shown. (E) 293T cells with stable human or feline HA-tetherin expression were transfected with 1.5 μg of the indicated FIVC36 proviral construct and analyzed as in Fig. 2A. This was repeated four times and a representative example is shown.

Although signal peptides do not show conservation of amino acid sequences, they are functionally tripartite, with a positively charged N-terminal segment of variable length and sequence, a single central hydrophobic core (the H region), and a short C-terminal and usually more conserved signal peptidase cleavage-site (19). In contrast to this typical architecture, we identified two distinct hydrophobic segments in Fsp, which we designated H1 and H2 (Fig. 4A and B). H1 is located in the central portion of Fsp, downstream of Rev exon 1 and upstream of the cleavage site-adjacent H2 motif. To test the involvement of these regions in tetherin antagonism function, as well as viral viability and the processing and trafficking of Env, we generated in-frame deletions in the full-length virus. Mutant ΔH2/ΔC deletes the traditional signal peptide hydrophobic motif and adjacent cleavage signal. Mutant Δ40 was designed to delete much of the region (40 amino acids) between the end of Rev first exon residues and the onset of the traditional hydrophobic signal peptide and cleavage signal. The Δ40 deletion spans most of the H1 hydrophobic motif (VFSILYLFTGYIVYFL) as well as a downstream region rich in charged (R, K, D, E) residues (Fig. 4A).

To assess the effects of partial Fsp deletion on FIV protein expression and replication, we examined budding and assembly in human 293T cells and spreading replication in feline CrFK cells. CrFK cells were used as they express undetectable amounts of endogenous tetherin, even after treatment with IFN-α (Fig. S1A), allowing us to specifically assess the effects of partial Fsp deletion on FIV replication in the absence of tetherin. In fact, FIVC36Δ40 retained Env expression and normal replication kinetics in CrFK cells, whereas the FIVC36ΔH2/ΔC mutant had much lower Env protein expression in cells and greatly diminished replication (Fig. 4C and D). Budding of wild-type FIV was inhibited by human but not feline tetherin as expected (Fig. 4E). In contrast, the Δ40 mutation had minimal difference in budding in the absence of tetherin but resulted in severe restriction in the presence of both feline and human tetherin (Fig. 4E). These results, combined with the above observations, confirm that Fsp confers resistance to domestic cat tetherin restriction of viral release from cells. Furthermore, the effects of Fsp on Env SU/TM expression and trafficking to the cell surface are separable from its tetherin antagonism function.

**FIG 5:**
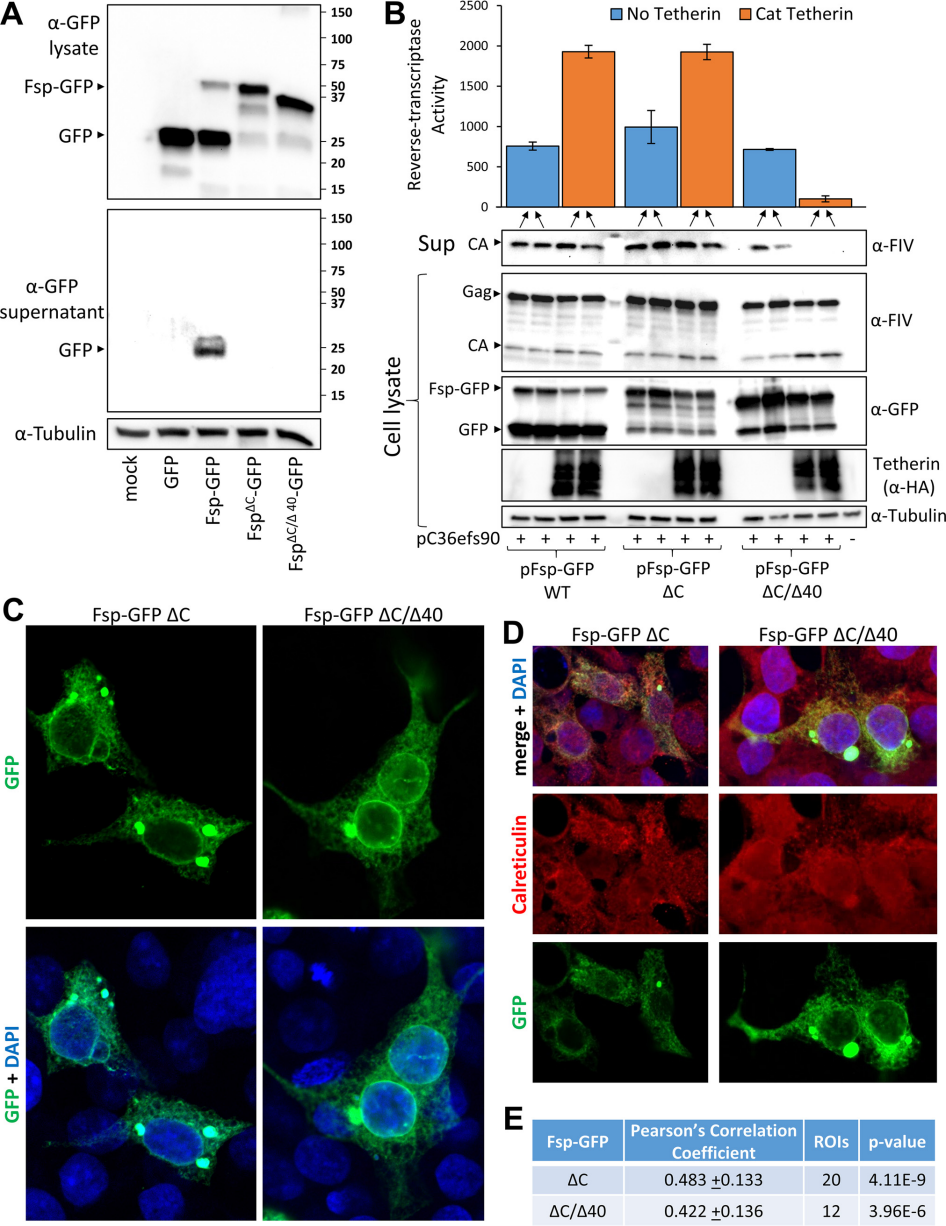
Fsp intracellular localization and tetherin counteraction when expressed in *trans*. (A) 293T cells were transfected with 1.5 μg of plasmids expressing eGFP or the indicated Fsp-GFP fusions. 24 h posttransfection cell lysates and 0.22 μm filtered supernatants were collected and immunoblotted with mouse anti-GFP or mouse anti-α-Tubulin and goat anti-mouse HRP. (B) Duplicate 293T wells were transfected with 1 μg of FIVC36efs90 and 500 ng pFsp-GFP plasmids. 24-h posttransfection cell lysates and 0.45 μm filtered supernatants were collected and immunoblotted with the indicated antibodies. Reverse-transcriptase activity is shown as the average of the biological duplicates +/− standard deviation. (C to E) Confocal microscopy of 293T cells transfected with pFsp-GFPΔC or pFsp-GFPΔC/Δ40 fixed 16 h posttransfection. Cells were mounted with ProLong Gold containing DAPI without additional staining (C) or were stained with rabbit anti-Calreticulin and Alexafluor 594 goat-anti-rabbit (D). (E) The ImageJ plugin Coloc2 was used to calculate Pearson’s Correlation Coefficient (+/− standard deviation) Fsp-GFP and Calreticulin as in Fig. 3F. A two-tailed *T*-test was performed to compare GFP-Calreticulin colocalization (Fig. 3D) with Fsp-GFPΔC or Fsp-GFPΔC/Δ40 colocalization with Calreticulin.

To more closely examine the intracellular localization of Fsp and its potential colocalization with tetherin, Fsp-GFP constructs were designed incorporating the ΔC mutant alone and in conjunction with the Δ40 deletion. Fsp-GFP lacking the C-region resulted in significantly weaker expression of cellular free GFP and no detectable supernatant accumulation of GFP than C-region intact Fsp-GFP, confirming that this region is the predominant target for signal peptidase enzymes (Fig. 5A). Moreover, an intact C-region was not required for Fsp-GFP to act *in trans* and enable efficient budding of Envelope deleted FIVC36 proviral constructs in domestic cat tetherin expressing cells, whereas the addition of the Δ40 deletion abrogated this particle budding effect (Fig. 5B). These results indicate that Fsp can function to counteract tetherin independently of other Env domains and is sufficient for FIV antagonism of tetherin.

Given that the Fsp-GFPΔC construct is functionally active and remains attached to GFP, we examined the subcellular localization of this protein and the ΔC/Δ40 mutant. Both proteins were cytoplasmic (Fig. 5C), and had similar colocalization with Calreticulin as the GFP signal from Fsp-GFP, with some perinuclear accumulation as in Fig. 3 (Fig. 3D and 5D). The colocalization of both Fsp-GFPΔC and Fsp-GFPΔC/Δ40 with Calreticulin is further evidence that after initial signal peptidase processing of the FIV Envelope Fsp becomes associated with the endoplasmic reticulum. The ability of Fsp to counteract domestic cat and canine tetherin but not human tetherin is suggestive of a direct interaction between these factors (11), yet we found there was not predominant colocalization between Fsp-GFPΔC and tetherin, nor was Fsp localization noticeably altered in the presence of tetherin (Fig. S3).

10.1128/mbio.00161-23.3 FIG S3 Fsp-GFP does not predominantly colocalize with tetherin. Confocal microscopy of 293T cells stably expressing HA-tetherin and transfected with pEGFP-N1, pFsp-GFP pFsp-GFPΔC, or pFsp-GFPΔC/Δ40. Cells were fixed and stained 16 h posttransfection with rat anti-HA (tetherin) and Alexafluor 594 goat-anti-rat. (B) The ImageJ plugin Coloc2 was used to calculate Pearson’s Correlation Coefficient (average +/− standard deviation shown) for GFP and Alexafluor594 signal as in Fig. 3 and 5 and a two-tailed *T*-test was calculated for each Fsp-GFP construct’s tetherin colocalization with respect to GFP. Download FIG S3, TIF file, 2.5 MB. Copyright © 2023 Morrison and Poeschla. 2023 Morrison and Poeschla. https://creativecommons.org/licenses/by/4.0/ This content is distributed under the terms of the Creative Commons Attribution 4.0 International license.

### Fsp acts by blocking tetherin incorporation into particles.

**FIG 6:**
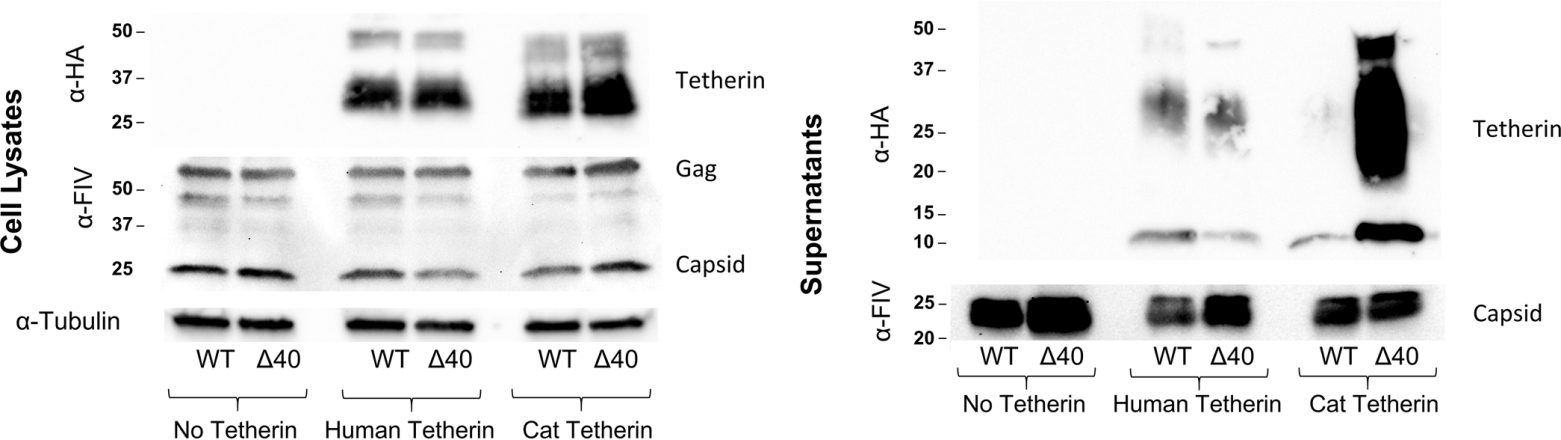
Fsp blocks virion incorporation of feline tetherin. Control and HA-tetherin expressing 293T cells were transfected with indicated FIV proviral constructs as in Fig. 2A. Supernatants were harvested and concentrated by ultracentrifugation over a 20% sucrose cushion. Concentrated virus was analyzed for reverse transcriptase activity and RT-normalized inputs were used for immunoblotting with the indicated antibodies. A representative example is show for this experiment, which was repeated at least four times with similar results.

Our previous data suggested that FIV antagonizes tetherin by a mechanism more similar to that of Ebola virus than that mediated by primate lentivirus accessory genes (11). Both viruses act in a way that is linked to their respective Env glycoproteins but does not reduce cell surface or intracellular tetherin levels (11, 20, 21) (see also Fig. 2A; Fig. S3). This observation suggests a mechanism that acts locally, on a per-particle basis at the point of viral budding, to exclude the factor from the particle (11). To determine whether Fsp alters the particle association of tetherin, wild-type or Δ40 FIV particles were produced in the presence or absence of stably expressed tetherins and then purified by ultracentrifugation over a sucrose cushion. Particles were then immunoblotted—using reverse transcriptase-normalized inputs—for tetherin. The results were dramatic. Wild-type FIVC36 virions contained minimal amounts of human or feline tetherin (Fig. 6). In contrast, FIVC36Δ40 virions contained similarly low levels of human tetherin, but high levels of feline tetherin (Fig. 6). Thus, the activity of Fsp is both powerful and specific: intact Fsp stringently blocks otherwise abundant virion incorporation of feline but not human tetherin. This mechanism is also unique among the lentiviral anti-tetherins.

## DISCUSSION

Our results reveal that the Env glycoprotein signal peptide is the FIV tetherin antagonist, thus identifying the fourth lentiviral anti-tetherin protein and also, to the best of our knowledge, identifying the first new lentiviral accessory protein in decades. Almost all of Env—all of SU and TM—was dispensable for tetherin antagonism. Fsp was both necessary and sufficient for enabling release of viral particles from tetherin expressing cells (Fig. 1 and 4). We identified a central 40 amino acid segment of Fsp that is located C-terminal to the first exon of Rev is not needed for Env processing, and the function of which was previously unknown. Deletion of the segment did not affect FIV replication in the absence of tetherin but severely impaired budding of the virus in its presence. The mechanism fulfills multiple criteria for a specifically evolved restriction factor antagonism, as it is also virus- and species-specific: Fsp did not protect FIV from human tetherin, or HIV-1 from either feline or human tetherin (Fig. 2 and 4E) (11).

Simple retroviruses encode *gag*, *pol*, and *env* genes that primarily encode the structural and enzymatic proteins needed for completing essential viral life cycle steps. Lentiviruses, by contrast, are complex retroviruses that establish persistent, lifelong infections and must therefore evade innate and adaptive immunity for years. To meet this challenge, they have evolved additional accessory proteins that modulate host immune responses and antagonize innate immune effectors. HIV-1 and simian lentiviruses variably use the small accessory proteins Vpu and Nef to overcome the block to nascent viral budding imposed by tetherin, which appears to have afforded genetic flexibility. The evolution of these proteins not only underlies lifelong persistence in individuals of a given species, but has also been shown to be critical for host switching, as in the case of the acquisition of anti-tetherin activity by Vpu during the adaptation of SIVcpz to become HIV-1 (6, 22). Presumably due to the pressures posed by such viral proteins and also from nonretroviral antagonists, tetherin proteins show evidence of positive selection in mammals (23–25).

Nonprimate lentiviruses encode more limited accessory gene repertoires than primate lentiviruses and they specifically lack Vpu and Nef proteins. The prior mapping of anti-tetherin activity to the *Env* gene of FIV suggested antagonism by the full Env glycoprotein similar to what has been observed for HIV-2 (10, 11, 26, 27). However, our data reveal that FIV SU and TM are dispensable. We showed previously that FIV does not degrade tetherin or downregulate it from the cell surface (11), identifying a contrast with primate lentiviral Vpu, Nef, or Env proteins, which all mediate functional depletion of tetherin from the primary site of viral budding via intracellular sequestration, endocytosis, and lysosomal degradation of the protein (8–10). Here, we show that Fsp instead excludes tetherin from the particle, establishing a mechanism unique among the lentiviruses (Fig. 6). Fsp possesses autonomous restriction blocking activity and can also direct the ER translocation and export of an unrelated protein (Fig. 3).

Signal peptides (signal sequences) act as intracellular zip codes that direct the location of many cellular proteins that are destined for extra-cytoplasmic locations. Proteins destined for secretion or plasma membrane residence are translocated as preproteins through or into the ER membrane. After preprotein translocation has partially completed, the signal peptidase enzyme cleaves the signal peptide away, which enables correct folding of the mature protein. Signal peptides are generally quite short (under 30 amino acids), do not have further functions, and are mostly degraded in short order by the signal peptide peptidase. In the case of RNA viruses, however, genome sizes are severely constrained and, for retroviruses, genetic efficiency in the form of overlapping reading frames and multiple purpose proteins, e.g., Nef, are observed. Dual purposing of the signal peptide by FIV is an interesting example. The amino acids encoded by the first exon of Rev are also present in the first 80 amino acids of Fsp, which adds a third function to this compressed region of the genome. There are a few prior examples of cellular protein signal peptides with additional cellular functions (28) and a few in other viruses as well. However, the latter generally involve direct participation in mechanics of the virus’s replication machinery. Following signal peptidase processing of the Arenavirus envelope glycoprotein, the signal peptide is not degraded and instead forms a tripartite complex with the mature glycoprotein subunits, which is necessary for glycoprotein mediated fusion with target cells (29). In this case the signal peptide function remains tied to that of the envelope protein. Among retroviruses, the spumaretrovirus foamy virus signal peptide binds to cognate Gag molecules and is packaged into viral particles, where it appears to be necessary for proper virion morphogenesis (30, 31). The signal peptides of the betaretroviruses mouse mammary tumor virus (MMTV) (32, 33) and Jaagsiekte sheep retrovirus (34) Env proteins traffic to the nucleoli of infected cells, where they are involved in modulating nuclear export of unspliced viral mRNAs.

In the case of Fsp, the signal peptide has evolved to counter a main host defense and can properly be considered a viral accessory protein. We propose that Env signal peptides with additional functions independent of directing Env translocation may in fact be a more general nonprimate lentivirus property, because the signal peptides of these viruses vary in length but are all significantly longer (66 to 176 amino acids; Fig. S2) than the signal peptides of primate lentiviruses and most cellular signal peptides (28, 35). Indeed, the full-length equine infectious anemia virus Env glycoprotein has been reported to counteract horse tetherin, also by a mechanism that does not degrade the factor; whether all or just part of Env is the antagonist has not been determined (36).

Our results underscore the centrality of tetherin to mammalian defense against lentiviruses in widely different circumstances. Paralleling the situation in human studies, the feline protein is inducible by type I IFN in primary lymphocytes (Fig. S1). We found it is abundant not only in spleen (FIV infects B and T cells *in vivo*), but also in lung, where it might play a role in restricting respiratory viruses. In regard to the substantial mRNA and protein levels in feline lung (Fig. S1B and C), human tetherin has been reported to inhibit SARS-CoV-2 budding (37). Feline and primate lentiviruses share distant ancestry, with the former likely to have colonized some but not all feline lineages sometime after the modern felid species radiation in the late Miocene, c.a. 11 million years ago (Mya) (38). Major commonalities do persist between FIV and HIV in pathophysiology (AIDS) and dependency factor utilization, such as CXCR4 and LEDGF, and both have Vif proteins that degrade APOBEC3 proteins (39–41). Domestic cat FIV is an AIDS-causing lentivirus like HIV-1, yet it and its ancestral felid species relatives have been on an independent evolutionary trajectory for millions of years. On the host side of the equation, the tiger and the domestic cat tetherin proteins share a likely more ancient (c.a. 60 to 11 Mya) truncation of the cytoplasmic tail, with the loss of 19 of 27 amino acids, including a dual tyrosine motif (11). The parallel evolution by FIV of an anti-tetherin protein that is structurally, functionally, and mechanistically very different from those of the primate lentivirus proteins in consistent with this and other evidence for the genetic plasticity of this host factor. The unusual architecture of tetherins rather than primary sequence is critical for their function, as well as their versatility against other groups of enveloped viruses (42–44). Investigation of other lentiviruses may uncover further viral solutions to the problem of tetherin.

## MATERIALS AND METHODS

### Cells, tissues, and animals.

In this study, 239T, Crandell feline kidney (CrFK), K-ER fibroblastic (45), and G355-5 astrocyte-derived cells were cultured in DMEM with 10% fetal calf serum (FBS), penicillin-streptomycin, and l-glutamine. Stable HA-tetherin-expressing cells have been previously described (11) and were additionally cultured in 3 μg/mL puromycin. All animal procedures were performed in accordance with the Guide for the Care and Use of Laboratory Animals (46) in an AAALAC-accredited facility and approved by the University of Colorado Institutional Animal Care and Use Committee (AAALAC Accreditation No. 00235; Protocol 00116). Felis catus tissues and PBMCs from control, uninfected animals were previously collected and described (47). Tissues pieces were placed into MagNA Lyser Green Beads with 0.75 mL TRIzol Reagent (Thermo Fisher Scientific) or 0.75 mL 1× radioimmunoprecipitation (RIPA) buffer (150 mM NaCl, 0.5% deoxycholate, 0.1% sodium dodecyl sulfate, 1% NP-40, 150 mM Tris-HCl pH 8.0) and homogenized in a Roche Magna Lyser (6,000 RPM, 60 s). mRNA was isolate following the TRIzol manufacturers protocol and cDNA was created using 5 μg RNA in a 20 μL total reaction with the Maxima H Minus cDNA Synthesis Master Mix. Tetherin (*BST-2*) and *GAPDH* qPCR was performed as previously described (11).

### Monoclonal mouse anti-cat tetherin antibodies.

Tetherin-specific mouse hybridomas were generated following vaccination of mice with Helicobacter pylori neutrophil-activating protein chimera (NAP). NAP chimera proteins significantly enhance immunogenicity of antigens and methods of protein expression, mouse immunization, and hybridoma isolation have been previously described (48–50). NAP-tetherin was cloned into the bacterial expression plasmid pET28a+ with His6 and MBP N-terminal tags for purification, separated by a TEV cleavage site. BL21(DE3) E. coli were transformed with the pET28 NAP-tetherin and His-tagged protein was purified with a NiNTA kit (Qiagen). Six- to 8-week-old BALB/c mice were intraperitoneal injected with 50 μg of purified NAP-tetherin with Freund’s adjuvant and boosted with 25 μg additional antigen by intravenous injection. Spleen cells were collected and fused with myeloma line Sp2/0-Ag14 (ATCC) using polyethylene glycol 4000. Hybridomas were screened for tetherin specific reactivity by antigen-mediated ELISA and immunoblotting. Two tetherin specific MAbs were identified, 34F11 and 35H2. Hybridoma supernatant was directly used as primary antibodies for experiments (1:20 to 1:50 dilution).

### Vectors, viruses, and plasmids.

pCT-C36^A+^ and pFE-C36 (subclone encoding Env protein) were used as the basis for mutagenesis (11). They employ the 5 ’U3-replacement strategy that enabled FIV production in human cells, in which the FIV U3 has virtually no promoter function (39, 51). In this case, we applied this to the proviral clone C36 (12), Additionally C36 *Env* was subcloned from NheI-digested pCT-C36^A+^ into the NheI site of gammaretroviral vector pJZ308 (39) to yield pJZC36. Env-frameshift mutants of C36 were constructed within pFE-C36 by site directed mutagenesis (Efs330) or overlap-extension PCR between NotI and MfeI restriction enzyme sites, approximately comprising *Env* amino acids 1 to 500. Insertions to frameshift Env are denoted here (lower-case letter indicates inserted nucleotide, enzyme in parentheses indicates restriction site added by nucleotide insertion):

Efs90: GGTAAGATATTTAAGATAtCTCTGATTTACAAGTATTTAG (EcoRV)

Efs134: CTGGGGAAAAATTTAatTAAAAATGAAAAGGGAC (PacI)

Efs176: GACAAGGTAAGGCACAAGctTAATATGGAGACTCCCACCC (HindIII)

Efs247: GAAAGCTACAAGAtAATcTAGAAGGGGAAAAGTTTGG (XbaI)

Efs330: CAAATCCCACTGATCAATTAgtcgacagTACATTTGGACCTAATC (ScaI)

Mutants were verified by restriction enzyme digestion and sequencing across the cloned fragment. An AvrII/BglII fragment from pFE-C36 was then moved into pJZ C36 (AvrII/BglII), then an NheI fragment from pJZ C36 was then swapped into an NheI digested pCT-C36 to create pCT-C36 mutants with the indicated insertions causing *Env* frameshift but no other changes. Each mutant was again verified by sequencing across the entire NheI fragment. C36Δ40 and C36ΔH2/ΔC were cloned by overlap extension PCR to remove 40 amino acids (residues 99 to 138) or 29 amino acids (ΔH2/ΔC, residues 150 to 178), and reinserted between AvrII and EcoNI digested pJZ C36, then an NheI fragment of pJZC36Δ40 or ΔH2 containing C36 *Env* was again inserted back into NheI digested pCT-C36^A+^. Codon optimized Fsp (amino acids 1–178) was synthesized as a gBlock (IDT). Complementary Fsp and GFP cDNAs were generated by PCR and cloned into a NotI/BglII digested p1012 (an expression plasmid having the human CMV immediate early promoter) (52) using the GeneArt Seamless Cloning System (Thermo Fisher Scientific), with a single amino acid linker (S) separating Fsp and GFP. Tetherin expression constructs Tsin HA-Tetherin (cat or human) have been previously described (11).

### Transfections and particle analyses.

A total of 4 × 10^5^ 293T cells were plated per well of a 6-well plate, allowed to adhere overnight, and PEI transfected. Then, 1.5 μg total DNA was added to 35 μL of Optimem without serum and 6 μL of 1 μg/μL PEI before brief vortexing and incubation at room temperature for 30 min. Transfection mix was added dropwise to cells and washed with fresh complete media after 8 to 16 h. After 48 h posttransfection, supernatant was harvested and filtered through a 0.45 μM filter. For analysis of tetherin incorporation into viral particles, supernatant was concentrated by ultracentrifugation over a 20% sucrose cushion. At the time of supernatant harvest, cells were lysed in 1× RIPA buffer. Immunoblotting was performed with cat serum reactive to FIV-PPR (gift of Peggy Barr), rat α-HA (Roche), mouse anti-GFP (JL-8 clone, TaKaRa Bio), or mouse anti-alpha-tubulin (Sigma). Immunoblot band density was quantified using ImageJ.

### Reverse-transcriptase activity.

Reverse-transcriptase (RT) activity was quantified by use of a real-time PCR assay as previously described (53). A total of 5 μL of supernatant was mixed with 5 μL of 2× viral lysis buffer (0.25% Triton X-100, 50 mM KCl, 100 mM TrisHCL, pH 7.4, 40% glycerol, and 2% vol/vol RNase inhibitor) and incubated at room temperature for 10 min, then 90 μL of sterile water was added. Samples were diluted 1:100 in sterile water, then 9 μL of diluted, lysed sample was used in a 20 μL qPCR containing 10 μL of 2× SYBR green master mix (Apex Sybr green, Quintarabio), 120 nM MS2 cDNA primers, and 0.055 A_260_ units of MS2 RNA (Sigma, catalog #10165948001).

### Immunofluorescence.

In total, 1 × 10^5^ 293T cells were plated on LabTek II chamber slides, allowed to adhere overnight and transfected with 500 ng indicated plasmids (pEGFP-N1 or pFsp-GFP). Next, 48 h posttransfection cells were fixed with 4% (wt/vol) paraformaldehyde for 10 min at room temperature, permeabilized with methanol, stained for 1 h at room temperature with rabbit anti-Calreticulin (1:50, Abcam ab2907) or anti-GORASP2 (1:100, Sigma HPA035274), washed 3× with PBS, and stained for 1 h at room temperature with Alexafluor 594-anti-rabbit-IgG (1:500). Wells were again washed 3× with PBS then mounted with ProLong Gold antifade reagent with DAPI (Invitrogen P36935). Images were collected on a Zeiss LSM780. For colocalization analysis, regions of interest were drawn around individual transfected cells where possible, or small groups of transfected cells and the ImageJ plugin Coloc2 was used to calculate Pearson’s correlation coefficients.

## References

[1] Neil SJ, Zang T, Bieniasz PD. 2008. Tetherin inhibits retrovirus release and is antagonized by HIV-1 Vpu. Nature 451:425–430. doi:10.1038/nature06553. 18200009

[2] Van Damme N, Goff D, Katsura C, Jorgenson RL, Mitchell R, Johnson MC, Stephens EB, Guatelli J. 2008. The interferon-induced protein BST-2 restricts HIV-1 release and is downregulated from the cell surface by the viral Vpu protein. Cell Host Microbe 3:245–252. doi:10.1016/j.chom.2008.03.001. 18342597 PMC2474773

[3] Miyakawa K, Ryo A, Murakami T, Ohba K, Yamaoka S, Fukuda M, Guatelli J, Yamamoto N. 2009. BCA2/Rabring7 promotes tetherin-dependent HIV-1 restriction. PLoS Pathog 5:e1000700. doi:10.1371/journal.ppat.1000700. 20019814 PMC2788703

[4] Cocka LJ, Bates P. 2012. Identification of alternatively translated tetherin isoforms with differing antiviral and signaling activities. PLoS Pathog 8:e1002931. doi:10.1371/journal.ppat.1002931. 23028328 PMC3460627

[5] Galao RP, Le Tortorec A, Pickering S, Kueck T, Neil SJ. 2012. Innate sensing of HIV-1 assembly by tetherin induces NFkappaB-dependent proinflammatory responses. Cell Host Microbe 12:633–644. doi:10.1016/j.chom.2012.10.007. 23159053 PMC3556742

[6] Sauter D, Schindler M, Specht A, Landford WN, Munch J, Kim KA, Votteler J, Schubert U, Bibollet-Ruche F, Keele BF, Takehisa J, Ogando Y, Ochsenbauer C, Kappes JC, Ayouba A, Peeters M, Learn GH, Shaw G, Sharp PM, Bieniasz P, Hahn BH, Hatziioannou T, Kirchhoff F. 2009. Tetherin-driven adaptation of Vpu and Nef function and the evolution of pandemic and nonpandemic HIV-1 strains. Cell Host Microbe 6:409–421. doi:10.1016/j.chom.2009.10.004. 19917496 PMC2779047

[7] Hirsch VM, Olmsted RA, Murphey-Corb M, Purcell RH, Johnson PR. 1989. An African primate lentivirus (SIVsm) closely related to HIV-2. Nature 339:389–392. doi:10.1038/339389a0. 2786147

[8] Jia B, Serra-Moreno R, Neidermyer W, Rahmberg A, Mackey J, Fofana IB, Johnson WE, Westmoreland S, Evans DT. 2009. Species-specific activity of SIV Nef and HIV-1 Vpu in overcoming restriction by tetherin/BST2. PLoS Pathog 5:e1000429. doi:10.1371/journal.ppat.1000429. 19436700 PMC2673686

[9] Zhang F, Wilson SJ, Landford WC, Virgen B, Gregory D, Johnson MC, Munch J, Kirchhoff F, Bieniasz PD, Hatziioannou T. 2009. Nef proteins from simian immunodeficiency viruses are tetherin antagonists. Cell Host Microbe 6:54–67. doi:10.1016/j.chom.2009.05.008. 19501037 PMC2852097

[10] Le Tortorec A, Neil SJ. 2009. Antagonism to and intracellular sequestration of human tetherin by the human immunodeficiency virus type 2 envelope glycoprotein. J Virol 83:11966–11978. doi:10.1128/JVI.01515-09. 19740980 PMC2772693

[11] Morrison JH, Guevara RB, Marcano AC, Saenz DT, Fadel HJ, Rogstad DK, Poeschla EM. 2014. Feline immunodeficiency virus envelope glycoproteins antagonize tetherin through a distinctive mechanism that requires virion incorporation. J Virol 88:3255–3272. doi:10.1128/JVI.03814-13. 24390322 PMC3957917

[12] de Rozieres S, Mathiason CK, Rolston MR, Chatterji U, Hoover EA, Elder JH. 2004. Characterization of a highly pathogenic molecular clone of feline immunodeficiency virus clade C. J Virol 78:8971–8982. doi:10.1128/JVI.78.17.8971-8982.2004. 15308694 PMC506922

[13] Blobel G, Dobberstein B. 1975. Transfer of proteins across membranes. I. Presence of proteolytically processed and unprocessed nascent immunoglobulin light chains on membrane-bound ribosomes of murine myeloma. J Cell Biol 67:835–851. doi:10.1083/jcb.67.3.835. 811671 PMC2111658

[14] Blobel G, Dobberstein B. 1975. Transfer of proteins across membranes. II. Reconstitution of functional rough microsomes from heterologous components. J Cell Biol 67:852–862. doi:10.1083/jcb.67.3.852. 811672 PMC2111655

[15] Blobel G. 2000. Protein targeting (Nobel lecture). Chembiochem 1:86–102. doi:10.1002/1439-7633(20000818)1:2<86::AID-CBIC86>3.0.CO;2-A. 11828402

[16] Hiss JA, Schneider G. 2009. Domain organization of long autotransporter signal sequences. Bioinform Biol Insights 3:189–204. doi:10.4137/bbi.s3411. 20072671 PMC2805444

[17] Pancino G, Fossati I, Chappey C, Castelot S, Hurtrel B, Moraillon A, Klatzmann D, Sonigo P. 1993. Structure and variations of feline immunodeficiency virus envelope glycoproteins. Virology 192:659–662. doi:10.1006/viro.1993.1083. 8380668

[18] Verschoor EJ, Hulskotte EG, Ederveen J, Koolen MJ, Horzinek MC, Rottier PJ. 1993. Post-translational processing of the feline immunodeficiency virus envelope precursor protein. Virology 193:433–438. doi:10.1006/viro.1993.1140. 8382405

[19] Owji H, Nezafat N, Negahdaripour M, Hajiebrahimi A, Ghasemi Y. 2018. A comprehensive review of signal peptides: structure, roles, and applications. Eur J Cell Biol 97:422–441. doi:10.1016/j.ejcb.2018.06.003. 29958716

[20] Lopez LA, Yang SJ, Hauser H, Exline CM, Haworth KG, Oldenburg J, Cannon PM. 2010. Ebola virus glycoprotein counteracts BST-2/Tetherin restriction in a sequence-independent manner that does not require tetherin surface removal. J Virol 84:7243–7255. doi:10.1128/JVI.02636-09. 20444895 PMC2898217

[21] Kuhl A, Banning C, Marzi A, Votteler J, Steffen I, Bertram S, Glowacka I, Konrad A, Sturzl M, Guo JT, Schubert U, Feldmann H, Behrens G, Schindler M, Pohlmann S. 2011. The Ebola virus glycoprotein and HIV-1 Vpu employ different strategies to counteract the antiviral factor tetherin. J Infect Dis 204 Suppl 3:S850–60. doi:10.1093/infdis/jir378. 21987761 PMC3189996

[22] Neil SJ. 2017. Exercising restraint. Cell Host Microbe 21:274–277. doi:10.1016/j.chom.2017.01.008. 28279329

[23] McNatt MW, Zang T, Hatziioannou T, Bartlett M, Fofana IB, Johnson WE, Neil SJ, Bieniasz PD. 2009. Species-specific activity of HIV-1 Vpu and positive selection of tetherin transmembrane domain variants. PLoS Pathog 5:e1000300. doi:10.1371/journal.ppat.1000300. 19214216 PMC2633611

[24] Lim ES, Malik HS, Emerman M. 2010. Ancient adaptive evolution of tetherin shaped the functions of Vpu and Nef in human immunodeficiency virus and primate lentiviruses. J Virol 84:7124–7134. doi:10.1128/JVI.00468-10. 20444900 PMC2898239

[25] Liu J, Chen K, Wang JH, Zhang C. 2010. Molecular evolution of the primate antiviral restriction factor tetherin. PLoS One 5:e11904. doi:10.1371/journal.pone.0011904. 20689591 PMC2912774

[26] Dietrich I, McMonagle EL, Petit SJ, Vijayakrishnan S, Logan N, Chan CN, Towers GJ, Hosie MJ, Willett BJ. 2011. Feline tetherin efficiently restricts release of feline immunodeficiency virus but not spreading of infection. J Virol 85:5840–5852. doi:10.1128/JVI.00071-11. 21490095 PMC3126296

[27] Celestino M, Calistri A, Del Vecchio C, Salata C, Chiuppesi F, Pistello M, Borsetti A, Palu G, Parolin C. 2012. Feline tetherin is characterized by a short N-terminal region and is counteracted by the feline immunodeficiency virus envelope glycoprotein. J Virol 86:6688–6700. doi:10.1128/JVI.07037-11. 22514338 PMC3393548

[28] Martoglio B, Dobberstein B. 1998. Signal sequences: more than just greasy peptides. Trends Cell Biol 8:410–415. doi:10.1016/s0962-8924(98)01360-9. 9789330

[29] Nunberg JH, York J. 2012. The curious case of arenavirus entry, and its inhibition. Viruses 4:83–101. doi:10.3390/v4010083. 22355453 PMC3280523

[30] Lindemann D, Pietschmann T, Picard-Maureau M, Berg A, Heinkelein M, Thurow J, Knaus P, Zentgraf H, Rethwilm A. 2001. A particle-associated glycoprotein signal peptide essential for virus maturation and infectivity. J Virol 75:5762–5771. doi:10.1128/JVI.75.13.5762-5771.2001. 11390578 PMC114292

[31] Geiselhart V, Schwantes A, Bastone P, Frech M, Lochelt M. 2003. Features of the Env leader protein and the N-terminal Gag domain of feline foamy virus important for virus morphogenesis. Virology 310:235–244. doi:10.1016/s0042-6822(03)00125-9. 12781711

[32] Dultz E, Hildenbeutel M, Martoglio B, Hochman J, Dobberstein B, Kapp K. 2008. The signal peptide of the mouse mammary tumor virus Rem protein is released from the endoplasmic reticulum membrane and accumulates in nucleoli. J Biol Chem 283:9966–9976. doi:10.1074/jbc.M705712200. 18270201

[33] Byun H, Halani N, Mertz JA, Ali AF, Lozano MM, Dudley JP. 2010. Retroviral Rem protein requires processing by signal peptidase and retrotranslocation for nuclear function. Proc Natl Acad Sci USA 107:12287–12292. doi:10.1073/pnas.1004303107. 20566871 PMC2901445

[34] Caporale M, Arnaud F, Mura M, Golder M, Murgia C, Palmarini M. 2009. The signal peptide of a simple retrovirus envelope functions as a posttranscriptional regulator of viral gene expression. J Virol 83:4591–4604. doi:10.1128/JVI.01833-08. 19244321 PMC2668452

[35] Pancino G, Ellerbrok H, Sitbon M, Sonigo P. 1994. Conserved framework of envelope glycoproteins among lentiviruses. Curr Top Microbiol Immunol 188:77–105. 7924431 10.1007/978-3-642-78536-8_5

[36] Yin X, Hu Z, Gu Q, Wu X, Zheng YH, Wei P, Wang X. 2014. Equine tetherin blocks retrovirus release and its activity is antagonized by equine infectious anemia virus envelope protein. J Virol 88:1259–1270. doi:10.1128/JVI.03148-13. 24227834 PMC3911658

[37] Martin-Sancho L, Lewinski MK, Pache L, Stoneham CA, Yin X, Becker ME, Pratt D, Churas C, Rosenthal SB, Liu S, Weston S, De Jesus PD, O'Neill AM, Gounder AP, Nguyen C, Pu Y, Curry HM, Oom AL, Miorin L, Rodriguez-Frandsen A, Zheng F, Wu C, Xiong Y, Urbanowski M, Shaw ML, Chang MW, Benner C, Hope TJ, Frieman MB, Garcia-Sastre A, Ideker T, Hultquist JF, Guatelli J, Chanda SK. 2021. Functional landscape of SARS-CoV-2 cellular restriction. Mol Cell 81:2656–2668. doi:10.1016/j.molcel.2021.04.008. 33930332 PMC8043580

[38] Pecon-Slattery J, Troyer JL, Johnson WE, O'Brien SJ. 2008. Evolution of feline immunodeficiency virus in Felidae: implications for human health and wildlife ecology. Vet Immunol Immunopathol 123:32–44. doi:10.1016/j.vetimm.2008.01.010. 18359092 PMC2774529

[39] Poeschla E, Looney D. 1998. CXCR4 is required by a non-primate lentivirus: heterologous expression of feline immunodeficiency virus in human, rodent and feline cells. J Virol 72:6858–6866. doi:10.1128/JVI.72.8.6858-6866.1998. 9658135 PMC109895

[40] Llano M, Saenz DT, Meehan A, Wongthida P, Peretz M, Walker WH, Teo W, Poeschla EM. 2006. An essential role for LEDGF/p75 in HIV integration. Science 314:461–464. doi:10.1126/science.1132319. 16959972

[41] Münk C, Beck T, Zielonka J, Hotz-Wagenblatt A, Chareza S, Battenberg M, Thielebein J, Cichutek K, Bravo IG, O'Brien SJ, Lochelt M, Yuhki N. 2008. Functions, structure, and read-through alternative splicing of feline APOBEC3 genes. Genome Biol 9:R48. doi:10.1186/gb-2008-9-3-r48. 18315870 PMC2397500

[42] Perez-Caballero D, Zang T, Ebrahimi A, McNatt MW, Gregory DA, Johnson MC, Bieniasz PD. 2009. Tetherin inhibits HIV-1 release by directly tethering virions to cells. Cell 139:499–511. doi:10.1016/j.cell.2009.08.039. 19879838 PMC2844890

[43] Heusinger E, Kluge SF, Kirchhoff F, Sauter D. 2015. Early vertebrate evolution of the host restriction factor tetherin. J Virol 89:12154–12165. doi:10.1128/JVI.02149-15. 26401043 PMC4645306

[44] Blanco-Melo D, Venkatesh S, Bieniasz PD. 2016. Origins and evolution of tetherin, an orphan antiviral gene. Cell Host Microbe 20:189–201. doi:10.1016/j.chom.2016.06.007. 27427209 PMC4989275

[45] Munk C, Zielonka J, Constabel H, Kloke BP, Rengstl B, Battenberg M, Bonci F, Pistello M, Lochelt M, Cichutek K. 2007. Multiple restrictions of human immunodeficiency virus type 1 in feline cells. J Virol 81:7048–7060. doi:10.1128/JVI.02714-06. 17459941 PMC1933292

[46] Institute of Laboratory Animal Resources (U.S.). Committee on Care and Use of Laboratory Animals. 2011. Guide for the care and use of laboratory animals. U.S. Dept. of Health and Human Services, Public Health Service, Bethesda, MD.

[47] Wongsrikeao P, Saenz D, Rinkoski T, Otoi T, Poeschla E. 2011. Antiviral restriction factor transgenesis in the domestic cat. Nat Methods 8:853–859. doi:10.1038/nmeth.1703. 21909101 PMC4006694

[48] Kohler G, Milstein C. 1975. Continuous cultures of fused cells secreting antibody of predefined specificity. Nature 256:495–497. doi:10.1038/256495a0. 1172191

[49] Iankov ID, Penheiter AR, Carlson SK, Galanis E. 2012. Development of monoclonal antibody-based immunoassays for detection of Helicobacter pylori neutrophil-activating protein. J Immunol Methods 384:1–9. doi:10.1016/j.jim.2012.06.010. 22750540 PMC3691681

[50] Iankov ID, Federspiel MJ, Galanis E. 2013. Measles virus expressed Helicobacter pylori neutrophil-activating protein significantly enhances the immunogenicity of poor immunogens. Vaccine 31:4795–4801. doi:10.1016/j.vaccine.2013.07.085. 23948230 PMC3903391

[51] Poeschla E, Wong-Staal F, Looney D. 1998. Efficient transduction of nondividing cells by feline immunodeficiency virus lentiviral vectors. Nat Med 4:354–357. doi:10.1038/nm0398-354. 9500613

[52] Vanegas M, Llano M, Delgado S, Thompson D, Peretz M, Poeschla E. 2005. Identification of the LEDGF/p75 HIV-1 integrase-interaction domain and NLS reveals NLS-independent chromatin tethering. J Cell Sci 118:1733–1743. doi:10.1242/jcs.02299. 15797927

[53] Vermeire J, Naessens E, Vanderstraeten H, Landi A, Iannucci V, Van Nuffel A, Taghon T, Pizzato M, Verhasselt B. 2012. Quantification of reverse transcriptase activity by real-time PCR as a fast and accurate method for titration of HIV, lenti- and retroviral vectors. PLoS One 7:e50859. doi:10.1371/journal.pone.0050859. 23227216 PMC3515444

[54] UniProt C. 2021. UniProt: the universal protein knowledgebase in 2021. Nucleic Acids Res 49:D480–D489. doi:10.1093/nar/gkaa1100. 33237286 PMC7778908

[55] Mertz JA, Simper MS, Lozano MM, Payne SM, Dudley JP. 2005. Mouse mammary tumor virus encodes a self-regulatory RNA export protein and is a complex retrovirus. J Virol 79:14737–14747. doi:10.1128/JVI.79.23.14737-14747.2005. 16282474 PMC1287593

