# Supplementary figures and images for: The Feline Immunodeficiency Virus Envelope Signal Peptide Is a Tetherin Antagonizing Protein
Source: mBio. 2023 Mar 16;14(2):e00161-23. doi: 10.1128/mbio.00161-23 (PMC10128041; doi:10.1128/mbio.00161-23)

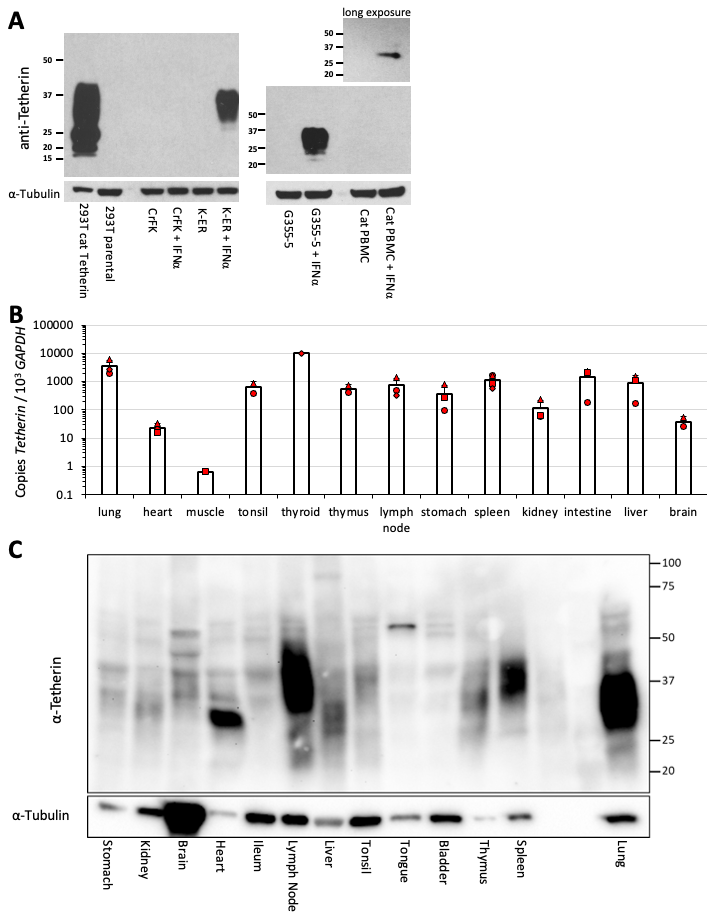

Supplement: FIG S1 [file mbio.00161-23-s0001.tif]

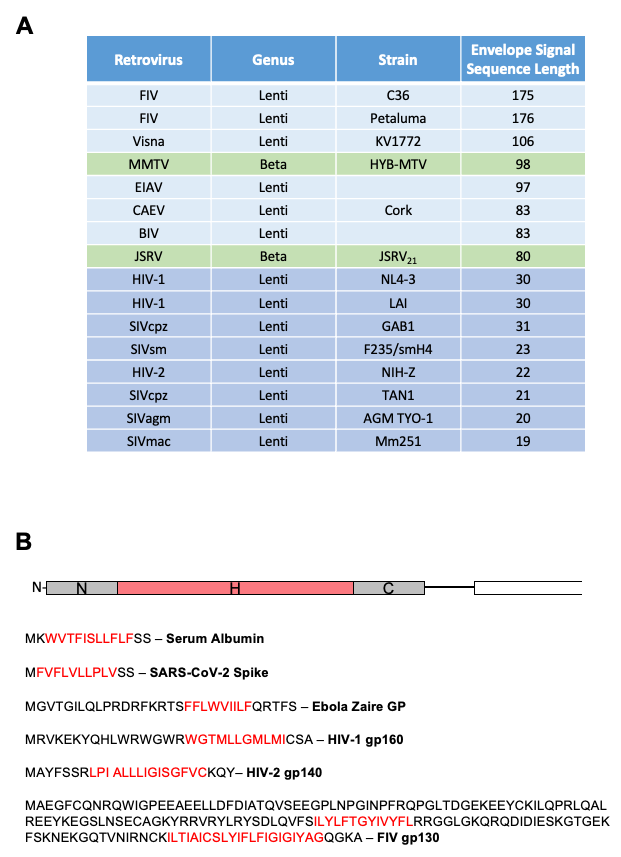

Supplement: FIG S2 [file mbio.00161-23-s0002.tif]

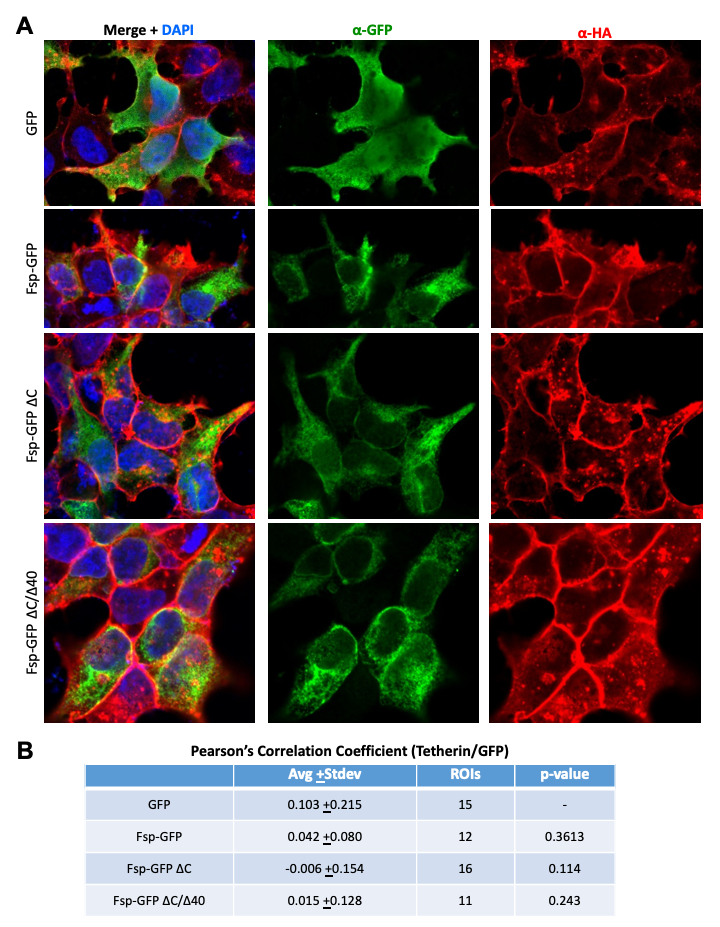

Supplement: FIG S3 [file mbio.00161-23-s0003.tif]
